# Expansion of human mesenchymal stem/stromal cells on temporary liquid microcarriers

**DOI:** 10.1002/jctb.6601

**Published:** 2020-11-08

**Authors:** Mariana P Hanga, Alvin W Nienow, Halina Murasiewicz, Andrzej W Pacek, Christopher J Hewitt, Karen Coopman

**Affiliations:** ^1^ Department of Biosciences, School of Life and Health Sciences Aston University Birmingham UK; ^2^ Centre for Biological Engineering, School of AACME, Chemical Engineering Department Loughborough University Loughborough UK; ^3^ School of Chemical Engineering University of Birmingham Birmingham UK; ^4^ Faculty of Chemical Technology and Engineering West Pomeranian University of Technology Szczecin Poland

**Keywords:** human bone marrow‐derived mesenchymal stem/stromal cells (hMSCs), temporary microcarriers, perfluorocarbon, scale‐up

## Abstract

**BACKGROUND:**

Traditional large‐scale culture systems for human mesenchymal stem/stromal cells (hMSCs) use solid microcarriers as attachment substrates. Although the use of such substrates is advantageous because of the high surface‐to‐volume ratio, cell harvest from the same substrates is a challenge as it requires enzymatic treatment, often combined with agitation. Here, we investigated a two‐phase system for expansion and non‐enzymatic recovery of hMSCs. Perfluorocarbon droplets were dispersed in a protein‐rich growth medium and were used as temporary liquid microcarriers for hMSC culture.

**RESULTS:**

hMSCs successfully attached to these liquid microcarriers, exhibiting similar morphologies to those cultured on solid ones. Fold increases of 3.03 ± 0.98 (hMSC1) and 3.81 ± 0.29 (hMSC2) were achieved on day 9. However, the maximum expansion folds were recorded on day 4 (4.79 ± 0.47 (hMSC1) and 4.856 ± 0.7 (hMSC2)). This decrease was caused by cell aggregation upon reaching confluency due to the contraction of the interface between the two phases. Cell quality, as assessed by differentiation, cell surface marker expression and clonogenic ability, was retained post expansion on the liquid microcarriers. Cell harvesting was achieved non‐enzymatically in two steps: first by inducing droplet coalescence and then aspirating the interface. Quality characteristics of hMSCs continued to be retained even after inducing droplet coalescence.

**CONCLUSION:**

The prospect of a temporary microcarrier that can be used to expand cells and then ‘disappear’ for cell release without using proteolytic enzymes is a very exciting one. Here, we have demonstrated that hMSCs can attach and proliferate on these perfluorocarbon liquid microcarriers while, very importantly, retaining their quality.

## INTRODUCTION

Human mesenchymal stem/stromal cells (hMSCs) are attractive candidates for large‐scale allogeneic therapy for multiple reasons: their availability in adults, relatively easy isolation from a variety of tissues, potential to differentiate to multiple cell types (adipocytes, osteocytes and chondrocytes), low immunogenicity and ability to induce regeneration by various mechanisms including exosomes and secretion of paracrine factors.^1^, ^2^ To date, hMSCs have been successfully cultured using attachment substrates such as microcarriers, which are usually solid particles, ~200–300 μm in size, made of a variety of materials, to provide a large surface area for growth and thus the potential for scale‐up rather than scale‐out;^3^ i.e., microcarriers allow hMSC cultures to be translated from traditional T‐flasks to bioreactor platforms. hMSCs have also been grown in both serum‐based and serum‐free media and at scales ranging from 15 mL in high‐throughput bioreactor platforms^4^ to spinner flasks,^5^, ^6^, ^7^, ^8^, ^9^ bench litre‐scale stirred tank reactors (STR)^10^, ^11^ and even up to 50 L.^12^

The microcarrier concept was first introduced by van Wezel in 1967, who used modified dextran.^13^ There are currently a variety of commercially available microcarriers with different surface chemistries to fulfil the needs of many types of cells. Microcarriers include those with the core made of alginate (GEM; Global Cell Solutions), collagen (CultiSpher G and S; Percell, Åstorp, Sweden), dextran (Cytodex 1 and 3; GE Healthcare, Chalfont St Giles, UK) or polystyrene (plastic, collagen, Hillex, ProNectin etc.; Pall SoloHill, Bingham Farms, MI, USA).^14^ The core material, surface properties and particle size are all important factors that influence cell adhesion and proliferation on such substrates. Thus commercially available microcarriers come in different sizes, with or without protein coating and with or without surface charge,^15^, ^16^ covering a wide range of applications and cell types. They also differ in porosity and density, which are important aspects to take into consideration when designing a bioprocess and planning the cell‐harvesting procedure.

Since hMSCs themselves are typically the product of a culture, particularly if intended for clinical use, they need to be detached and separated from the attachment substrate. In static planar conditions, this process is simple as it comprises one step alone, namely the incubation of the cells with a proteolytic enzyme such as trypsin.^7^ However, in stirred bioreactors with microcarriers, harvesting is more complex. It involves two steps: first, cell detachment from the microcarriers to produce a cell–microcarrier suspension; and, second, a cell separation step to separate the cells from the microcarriers. Cell detachment from microcarriers just by enzymatic means proved inefficient in spinner flasks and had to be combined with agitation,^17^ with the cell separation from microcarriers being achieved by filtration. Subsequently, the detachment technique was applied to other stirred bioreactors and microcarriers.^18^ To date, this approach has continued to work well,^19^ but there is always the possibility with some donor cells that such agitation may impact cell yield or quality. The overall approach increases in complexity with the increase in scale, and nothing has been published to substantiate its effectiveness at scale. In addition, there are concerns that enzymatic treatments for cell harvest could result in proteome alterations,^20^ which can raise safety issues when using cells for therapies. As such, a non‐enzymatic harvesting procedure would be preferable and it is that topic which this paper addresses.

Non‐enzymatic approaches for cell harvest from microcarriers have previously been investigated by utilising specifically designed microcarriers such as temperature‐responsive^21^, ^22^, ^23^ or dissolvable^24^ ones. Temperature‐responsive microcarriers are capable of non‐enzymatic cell harvest by manipulating the culture temperature, which determines a gradual change in hydrophilicity, leading to a gradual cell release. Although this approach has some advantages, it requires engraftment of the temperature‐responsive polymer on existing microcarriers, which needs specialist knowledge and/or equipment. In addition, careful consideration of the impact of the required culture temperature change on cell quality is necessary.

Another type of such microcarrier is dissolvable and uses modified hydrogels, but they still require a specific enzymatic treatment to dissolve the microcarrier and thus to release the cells without breaking the bonds proteolytically. Rodrigues *et al*. tested the Corning dissolvable microcarriers and found them successful at expanding induced pluripotent stem cells, but more importantly at recovering them from the microcarriers with a 92% harvesting yield.^24^ However, the Corning dissolvable microcarriers have now been discontinued, which means that preparation of such microcarriers again requires specific skills and expertise.

**Figure 1:**
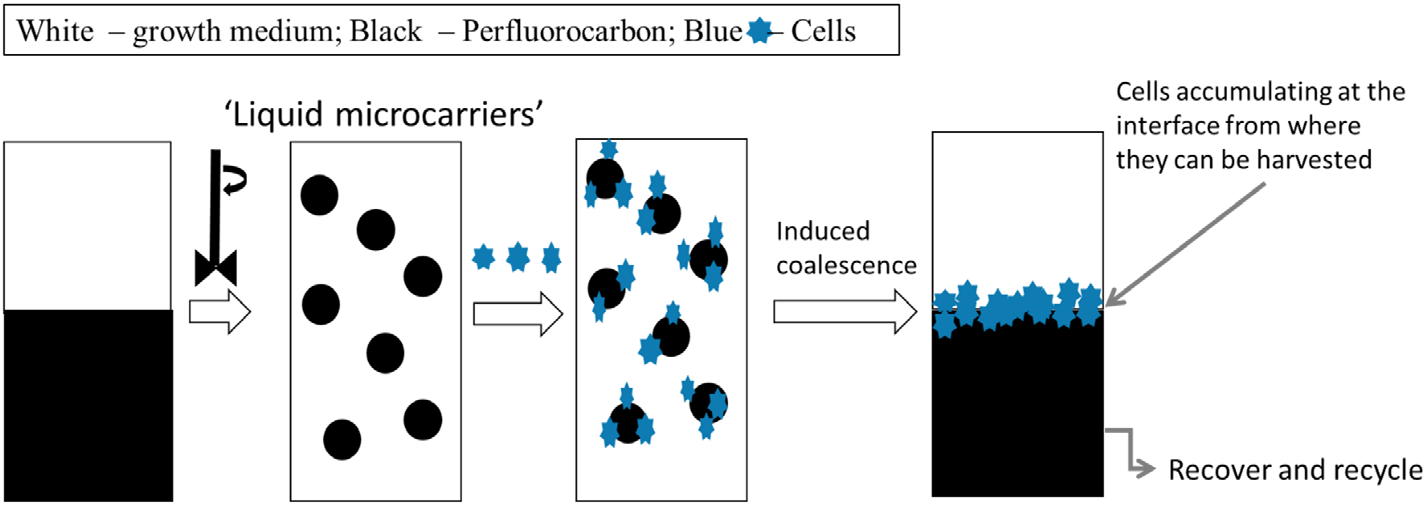
Schematic for the production of temporary microcarriers. The perfluorocarbon is mixed with growth medium to form a dispersion of stable liquid perfluorocarbon droplets that can be used as temporary microcarriers. The cells can be inoculated and expanded on to these liquid microcarriers. When harvest is required, coalescence of the droplets is induced, resulting in phase separation with accumulation of cells at the interface, from where they can be readily collected. The perfluorocarbon can then be recovered and recycled.^25^

The concept of a temporary microcarrier based on an immiscible oil phase in an agitated STR is an exciting prospect that could address all of these challenges and result in a simplified bioprocess. It would allow for cell expansion on the drops generated by stirring and, when required at the end of the expansion phase, cell harvest could be achieved non‐enzymatically by inducing droplet coalescence. If coalescence resulted in cell accumulation at the interface between the two phases, it should be possible to readily collect the cells from it by centrifugation or aspiration (Fig. 1). Thus this technology would not require either a detachment or a filtration step for separation of microcarriers, resulting in a simplified bioprocess. The temporary liquid microcarrier concept explored here comprised a two‐phase system of a selected perfluorocarbon (Fluorinert FC40) and protein‐rich growth medium. This system was previously tested in a planar set‐up and found successful for the expansion of hMSCs, while retaining cell quality.^25^ Moreover, the prospect of recycling the perfluorocarbon was also tested in our previous study,^25^ with no significant difference found in cell expansion on fresh or recycled perfluorocarbon. This finding is important as it is anticipated that recycling the perfluorocarbon, particularly at large scales, could lead to a reduction of cost associated with the use of substrates for cell attachment.

## MATERIALS AND METHODS

### Monolayer expansion of hMSCs

Two hMSCs, referred to here as hMSC1 and hMSC2, were isolated from bone marrow aspirates (Lonza, Cologne, Germany) from two healthy donors after informed consent. For all experiments, hMSCs between passages 2–6 were used. The cells were cultured according to previously determined protocols.^7^, ^25^ Briefly, the cells were seeded at 5000 cells cm^−2^ and cultured on tissue culture plastic in growth medium comprising DMEM (1 g L^−1^ glucose, Lonza) supplemented with 10% (v/v) foetal bovine serum (HyClone, Thermo Fisher, Waltham, MA, USA) and 2 mmol L^−1^ UltraGlutamine (Lonza, Castleford, UK). Growth medium was replaced every 3 days and the cells were passaged every 6 days when 70–80% confluency was achieved. Cell subculture was done by incubating with 0.25% (w/w) trypsin/EDTA for 7 min at 37 °C in a humidified incubator. The enzyme was then inactivated by the addition of pre‐warmed growth medium and the cell suspension was centrifuged at 220 × *g* for 5 min at room temperature. The obtained cell pellet was then resuspended in a known volume of medium for cell counts and further processing.

### Preparation of liquid microcarriers

The perfluorocarbon selected for preparing the liquid microcarriers was Fluorinert FC40 (3M, Sigma Aldrich, UK). This perfluorocarbon was found to promote attachment and proliferation of hMSCs, while retaining their quality post expansion when used as a 2D planar culture system.^25^ To prepare the liquid microcarriers, an emulsion of Fluorinert FC40 perfluorocarbon and growth medium was made by mixing the two components. Initially, the perfluorocarbon was sterilised by autoclaving at 121 °C and 2 atm for 15 min. For all experiments, ultralow attachment well plates (Corning, Hawarden, UK) were used to ensure cell attachment was only achieved on the liquid microcarriers and not on the tissue culture plastic. For the preparation of liquid microcarriers, pre‐sterilised perfluorocarbon needs to be dispersed in the growth medium (containing serum). This dispersion could be achieved by mixing, using various methods including an impeller directly in the bioreactor vessel.^26^ In this study, because of the small quantities needed, the dispersion of perfluorocarbon in serum‐containing medium was done by vortexing for 30 s (SciQuip vortex mixer). A 1:5 perfluorocarbon‐to‐growth medium ratio (equivalent of a 16.7% volume fraction) was used to prepare the liquid microcarriers. Droplets with a mean diameter (*d*_0.5_) of 292 μm and a span value below 1 were produced (Table 1). Span values of less than the unity are considered uniform size distributions.^27^, ^28^ Prior to cell inoculation, the liquid microcarriers were conditioned in growth medium for at least 1 h at 37 °C and 5% CO_2_ in a humidified incubator to allow proteins from the serum present in the medium to deposit onto the liquid microcarriers to promote cell attachment and proliferation.

**Table 1:** Perfluorocarbon/DMEM emulsion droplet size analysis based on volumetric size distribution

Perfluorocarbon‐to‐growth medium ratio	*D*(0.1)/μm	*D*(0.5)/μm	*D*(0.9)/μm	*D*(3.2)/μm	Span
1:5	191.73	292.62	444.36	277.45	0.863

### Cell culture on the temporary liquid microcarriers

The equivalent of 12 cm^2^ of interfacial surface area provided by the liquid droplets was inoculated into each well in ultralow attachment well plates (Corning). The interfacial surface area per volume of emulsion was calculated using Eqns (1) and (2) below:

(1) $$\text{Interfacial area}\;\mathrm{per}\;\text{volume}=\frac{6*\text{Volume fraction of dispersed phase}}{d_{32}}\;\left[m^{2}\;m^{-3}\right]$$

(2) $$\text{Volume fraction of dispersed phase}=\frac{V_{i}}{V_{\text{total}}}$$

where *d*_32_ represents the Sauter diameter [m^3^ m^−2^], *V*_*i*_ is the volume of dispersed phase [m^3^] and *V*_total_ is the total volume of dispersion produced in one batch [m^3^]. The Sauter diameter was obtained by measuring the droplet size and volumetric size distribution using a laser diffraction particle analyser (Mastersizer 2000, Malvern Instruments, Great Malvern, UK).

The hMSCs were inoculated on to the liquid microcarriers at 10 000 cells cm^−2^ of interfacial area and were kept in culture for up to 6 days. To avoid removing liquid microcarriers and attached cells, an 80% medium exchange was performed every 2 days. Experiments were performed under static conditions only and in ultra‐low attachment well plates. The well plates were kept in a humidified incubator at 37 °C and 5% CO_2_.

### Cell harvest

The perfluorocarbon droplets dispersed in the protein‐rich medium were found to be highly stable even though no stabilising reagents (e.g. surfactants) were added. This stability was investigated in one of our previous studies,^26^ where we hypothesised that it is caused by the proteins present in the media. Similarly, another hypothesis was that by removing the proteins present through repeated washes the droplets would then be destabilised and coalescence would be induced^26^ to release the cells. To test that hypothesis, the cells cultured on the liquid microcarriers were harvested by two methods: (i) by incubating with 0.25% trypsin–EDTA at 37 °C and 5% CO_2_ for 7 min in a humidified incubator; or (ii) by inducing droplet coalescence through repeated washes with Dulbecco's phosphate‐buffered saline (D‐PBS) (without calcium and magnesium), leading to non‐enzymatic cell release and accumulation to the interface, from where the cells were then collected by aspiration using a sterile Pasteur pipette. The cell pellet was then obtained by centrifugation at 220 × *g* for 5 min.

### Cell characterisation

hMSCs were characterised before and after expansion on the liquid microcarriers and after harvest by differentiation towards the three lineages (adipogenic, osteogenic and chondrogenic), by assessing clonogenic potential, by multiparameter flow cytometry to assess the expression of a panel of five cell surface markers and by sandwich ELISA to measure production of selected pro‐angiogenic growth factors. Cells cultured on tissue culture polystyrene (TCPS) were used as controls.

#### *Trilineage differentiation*

All chemicals were purchased from Sigma Aldrich unless otherwise stated. hMSCs were differentiated towards adipogenic, osteogenic and chondrogenic lineages using StemPro differentiation kits (Thermo Fisher). A 12‐well plate was used for all experiments. hMSCs were seeded at 5000 cells cm^−2^ for osteogenic differentiation and 10 000 cells cm^−2^ for adipogenic differentiation. For chondrogenic differentiation, the micromass method was used.^7^ Briefly, 5 μL droplets of a highly concentrated cell suspension (1 × 10^7^ cells mL^−1^) were seeded in an empty well plate and allowed to attach for 1–2 h at 37 °C in an incubator. Upon cell attachment, chondrogenic differentiation medium (StemPro) was added. For all differentiation cultures, the cells were grown in their respective media for 21 days, with a medium change performed every 3–4 days. At the end of 21 days, the cells were fixed with 4% paraformaldehyde for 20 min at room temperature. Adipocytes were stained with 0.3% (v/v) Oil Red O in 99% isopropanol for 10 min at room temperature, followed by two washes with distilled water. Chondrocytes were stained with 1% (v/v) Alcian Blue in 0.1 mol L^−1^ HCl solution for 60 min. Osteogenic differentiation potential was assessed using von Kossa stain, as previously described.^25^ Briefly, the cells were first stained for alkaline phosphatase (ALP) at room temperature in the dark for 45 min, followed by silver nitrate incubation at room temperature in the presence of light for 30 min to stain bone mineralisation. Imaging was carried out on a Nikon Ti Eclipse phase contrast microscope.

#### *Clonogenic potential*

Two hundred and fifty cells were inoculated into growth medium in a T25 flask. A minimum of five flasks were inoculated and placed in a humidified incubator set at 37 °C and 5% CO_2_. The medium was changed every 3–4 days and the culture was carried out for up to 14 days. The colonies were fixed and stained with 1% crystal violet (Sigma Aldrich, UK) for 30 min at room temperature and then manually counted. Colony‐forming unit fibroblast (CFU‐f). efficiency was calculated using Eqn (3):

(3) $$\mathrm{CFU}-f\;\text{efficiency}=\frac{\text{Number of colonies counted}}{\text{Number of cells seeded}}\times 100\;\left[\%\right]$$

#### *Immune‐phenotype assessment*

The immune‐phenotype of the hMSCs used was assessed by multi‐parameter flow cytometry using a previously developed protocol.^29^ Briefly, the cells were harvested from the substrate as described previously and counted, and the cell suspension was concentrated to achieve a concentration of 1 × 10^6^ cells mL^−1^. 200 μL of cell suspension was then loaded on to a V‐bottom 96‐well plate and centrifuged at 220 × *g* for 5 min. The supernatant was aspirated, and the cell pellet was resuspended in cell staining buffer (R&D Systems, Abingdon, UK) and centrifuged again. The cells were stained with mouse anti‐human monoclonal antibodies (BD Biosciences, Swindon, UK) for 30 min in the dark at room temperature. The fluorescently labelled antibodies were selected based on a panel recommended by the International Society for Cell Therapy (ISCT);^30^ these were CD73 (PE‐Cy7), CD90 (APC), CD105 (PE), CD34 (PE‐Cy5) and HLA‐DR (FITC). The stained samples were analysed on a Guava EasyCyte 8HT flow cytometer (Merck Millipore, Watford, UK) equipped with 488 nm and 640 nm excitation lasers. A minimum of 10 000 gated events were recorded for each sample. Post‐acquisition analysis and compensation were performed using FlowJo software v8 (Treestar Inc., Meza, AZ, USA).

#### *Assessment of pro‐angiogenic growth factors secretion profile*

Conditioned medium from hMSCs cultured on TCPS and on liquid microcarriers at different time points in culture was collected and analysed using the multiplex assay Luminex Magpix kits (R&D Systems) according to the manufacturer's instructions. The kits were customised to include the following factors: IL8, basic fibroblast growth factor (bFGF), hepatocyte growth factor (HGF), vascular endothelial growth factor (VEGF) and platelet‐derived growth factor (PDGF‐AA). Standards were prepared by reconstituting a premixed cocktail. Capture microparticle cocktail was added to each well of a 96‐well assay plate. Samples or standards prepared in triplicate were added to each well, sealed in foil and incubated for 2 h at room temperature on an orbital shaker. The plate was washed three times, incubated with biotin antibody cocktail for 1 h at room temperature, then washed and incubated with streptavidin–phycoerythrin for a further 30 min. Beads were captured and analysed using a BioPlex Magpix Multiplex reader. Array data were quantified using BioPlex Data Pro software.

### Analytical techniques

Droplet size and volumetric size distribution were measured using a Mastersizer 2000 (Malvern Instruments) laser diffraction particle analyser. The polydispersity of the droplet sizes was calculated as the span, which is the width of distribution based on 10%, 50% and 90% of the cumulative distribution of sizes:

(4) $$\text{Span}=\frac{D\left(0.9\right)-D\left(0.1\right)}{D\left(0.5\right)}$$

<NI>where *D*(0.1), *D*(0.5) and *D*(0.9) are droplet sizes at which 10%, 50% and 90%, respectively, of the volume distribution lies on the cumulative curve.

Cell morphology was assessed by phase contrast on a Nikon Ti Eclipse microscope. Cell counts were performed using a Nucleocounter NC‐3000 (Chemometec) directly on the liquid microcarriers using the reagent A100 and reagent B protocol. Briefly, the cell–microcarrier suspension was diluted in a 1:3 ratio with reagent A100 (lysing agent) and then reagent B (stabilising agent). The resulting suspension was then loaded onto a *Via*‐1 cassette containing acridine orange and DAPI.

Based on cell counts, the fold increase was calculated using the equation below:

(5) $$\mathrm{FI}=\frac{\mathrm{Cx}\left(f\right)}{\mathrm{Cx}\left(0\right)}$$

<NI>where *Cx*(f) represents the maximum cell number and *Cx*(0) is the initial cell number.

### Statistical analysis

All experiments were performed in triplicate as a minimum. Cell counts for every time point were acquired from two independent samples from each replicate. Data were expressed as mean ± SD. Statistical analysis was carried out using GraphPad Prism 8 software. For comparison between two data sets, statistical significance was determined by using Student's two‐tailed *t*‐test. For comparison of multiple data sets, significance was calculated by the one‐way or two‐way ANOVA test. Significance was determined at *P* < 0.05.

## RESULTS AND DISCUSSION

The aim of this work was to show that: (i) perfluorocarbon droplets could be used as liquid microcarriers for the expansion of hMSCs; (ii) non‐enzymatic cell harvest could be achieved (i.e., test the temporary microcarrier concept); and (iii) the cells retained their quality attributes both post‐expansion and post‐droplet coalescence (allow a non‐enzymatic cell harvest).

### Expansion of hMSCs on the liquid microcarriers

A size range comparable to commercially available solid microcarriers was provided by the 1:5 ratio (16.7% volume fraction) of perfluorocarbon‐to‐growth medium. As such, this formulation was taken forward to be tested as temporary liquid microcarriers for the growth of hMSCs. A previous study in planar culture^25^ showed that hMSCs were able to attach and proliferate at the interface between the same perfluorocarbon (Fluorinert FC40) and growth medium, while exhibiting a similar cell morphology to hMSC cultured on TCPS. Moreover, the same study demonstrated that hMSCs retained their ability to differentiate towards the three lineages, their clonogenicity potential and expression of cell surface markers. As such, a similar behaviour was expected when inoculating hMSCs on to the liquid perfluorocarbon microcarriers.

**Figure 2:**
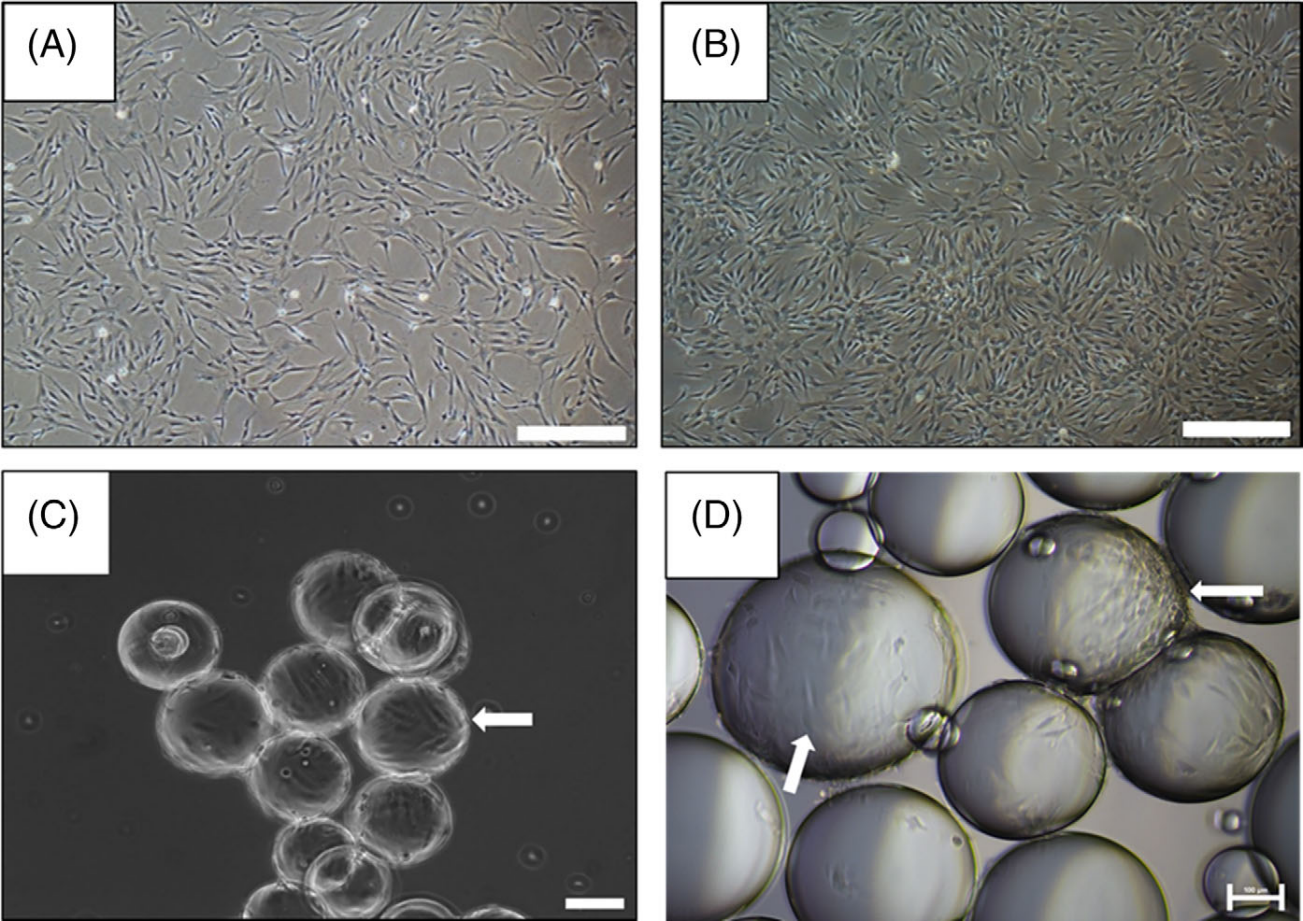
hMSC morphology when cultured on: (A) tissue culture plastic; (B) 2D perfluorocarbon/DMEM system; (C) commercially available microcarriers (e.g., Cytodex‐1); and (D) perfluorocarbon ‘liquid microcarriers’. Scale bar represents, in (A) and (B), 500 μm; and, in (C) and (D), 100 μm. White arrows show the elongated shape of hMSCs on the Cytodex solid microcarriers (C) and the perfluorocarbon liquid microcarriers (D).

Cell morphology was visually assessed. Figure 2(A) shows hMSC2 donor line morphology when cultured on TCPS, while Fig. 2(B) shows the morphology of the same donor hMSC line when cultured on the perfluorocarbon/growth medium interface in a planar set‐up. Microcarriers are curved surfaces and, as a result, cell morphology can appear slightly different to that on planar surfaces. A transparent microcarrier (e.g., Cytodex 1, GE Healthcare) (Fig. 2C) was chosen to enable a better visualisation of cell morphology for comparison to the liquid microcarriers (Fig. 2D). Elongated cell morphologies, typical to hMSCs, were observed on both types of microcarriers – solid (Fig. 2C) and liquid (Fig. 2D), as indicated by the white arrows.

**Figure 3:**
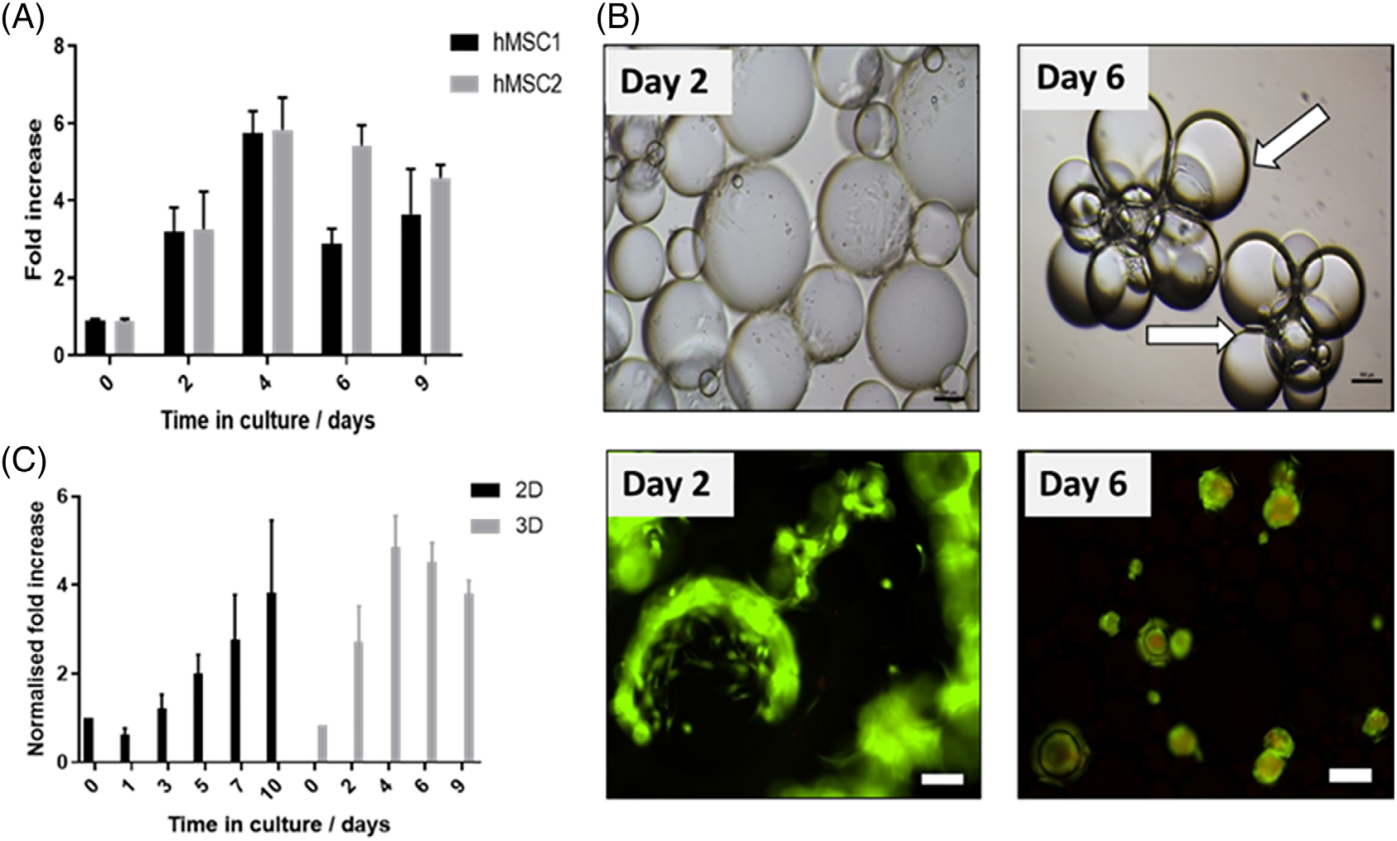
(A) Fold increase over 9 days in static culture on liquid microcarriers for two hMSC lines; data are expressed as mean ± SD (*n* = 4). (B) Phase contrast and live/dead merged images of hMSCs on liquid microcarriers at day 2 and day 6 in culture. Scale bars represent 100 μm. (C) Normalised fold increase over time in culture for hMSC2 donor line when cultured on either the 2D or the 3D perfluorocarbon/DMEM system; data are shown as mean ± SD (*n* = 4).

Two hMSC donor lines (hMSC1 and hMSC2) were cultured for a period of 9 days on the liquid microcarriers (Fig. 3A). An increase in cell number was recorded up to day 4 in culture; however, beyond this point, a decrease was observed. As the cells grew and occupied more of the surface area of each liquid microcarrier, the protein layer deposited on the interface between the two phases became stretched, thus resulting in altering the shape of the liquid microcarriers from spherical to tear‐shaped droplets (Fig. 3B). Moreover, as the cells grow, with conventional microcarriers, the tendency is for cells to form bridges between them.^9^ However, on the liquid microcarriers, bridging resulted in formation of cell aggregates at the centre of a droplet bouquet as indicated in Fig. 3(B) by the white arrow. The formation of the droplet ‘bouquet’ was observed only after 4 days, indicating that confluency on some microcarriers was achieved at that time point, while others had barely any cells attached. These experiments were carried out under static conditions, which explains the heterogenic distribution of cells on the liquid microcarriers. Moreover, a high cell seeding density (10 000 cells cm^−2^) was used for inoculation that was two times higher than the seeding density used on solid microcarriers in our previous studies.^4^, ^6^, ^9^ A lower cell seeding density could result in confluency being reached more slowly and an overall increase in fold expansion being achieved. However, this study was only a proof‐of‐concept demonstration and not an optimised expansion bioprocess. Live/dead staining showed the maintenance of cell viability, with an increase in dead cells towards the later stages of culture (day 6) localised in the centre of the aggregates formed (Fig. 3B). The formation of aggregates and the reaching of confluency can explain the decrease in cell number from day 4 to day 9 of culture (Fig. 3A). A sharper decrease of approximately 50% in cell number was observed for hMSC1 donor line, compared to only approximately 10% for hMSC2 donor line. This difference could be attributed to donor‐to‐donor variability.^7^

The growth of the same hMSC2 donor line was measured over time in culture on both the 2D planar and liquid microcarriers formed with Fluorinert FC40 and growth medium. When compared, cell growth on the liquid microcarriers achieved a fold increase normalised to the surface area of 3.81 ± 0.25 at day 9, while 3.83 ± 1.41 at day 10 was achieved on the planar interface (2D). However, the highest normalised fold increase on the liquid microcarriers was achieved at day 4 (4.86 ± 0.61) (Fig. 3C). After this time point, a decrease in cell number was recorded similarly to Fig. 3(A) and most likely for the same reasons. The improved growth on liquid microcarriers in a proof‐of‐concept demonstration is highly promising as the production of large numbers of stem cells on an immiscible liquid planar surface suffers from the same limitations as growth in standard T‐flasks.

### Cell quality assessment pre and post expansion on liquid microcarriers

**Figure 4:**
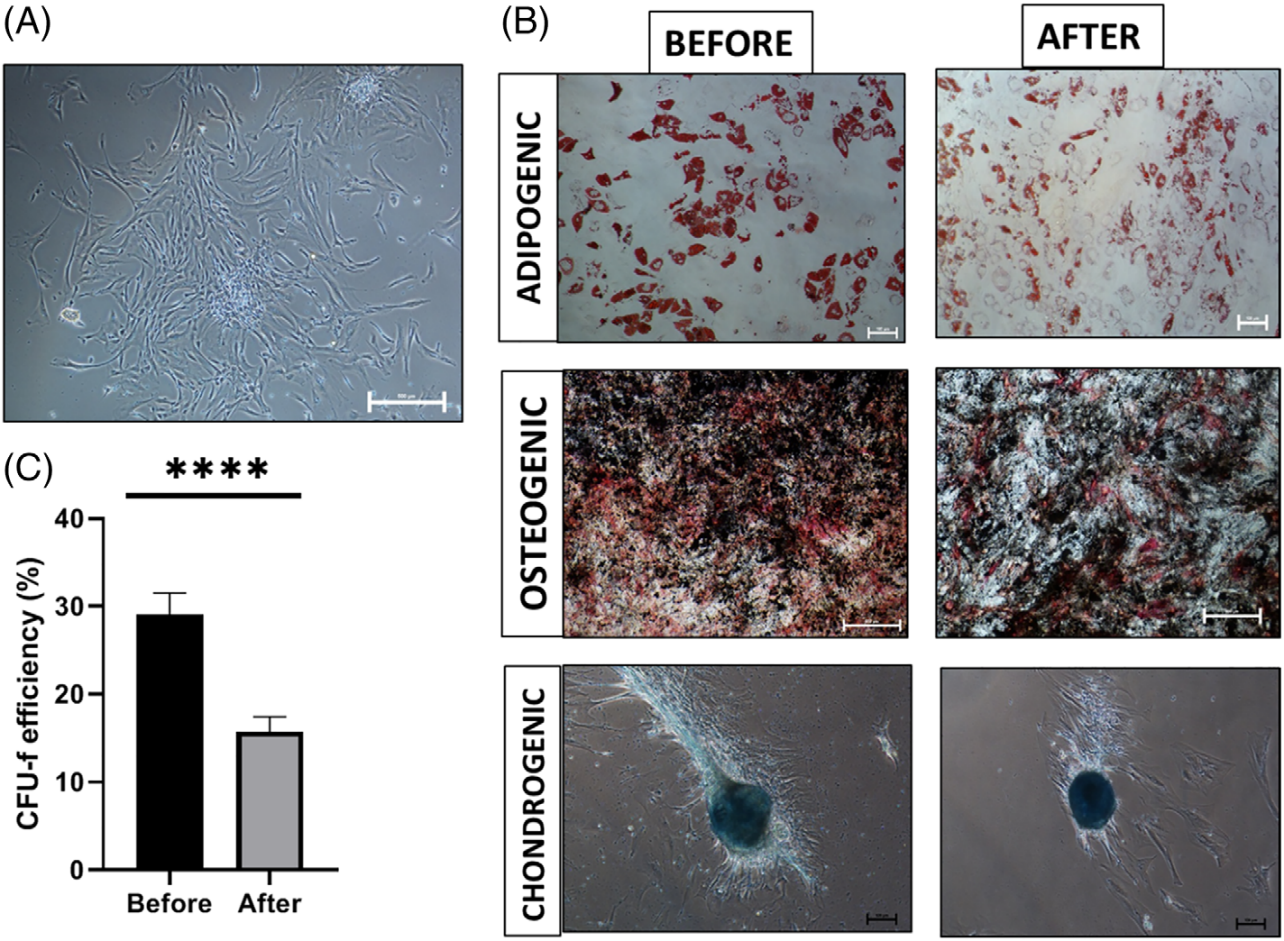
hMSC2 donor line quality assessment post expansion on the liquid microcarriers. (A) Cell morphology after replating on TCPS; scale bar represents 500 μm. (B) Differentiation to the three lineages before and after culture on liquid microcarriers; scale bars for adipogenic images 100 μm, and for osteogenic images 500 μm. (C) CFU‐f efficiency; data are shown as mean ± SD (*n* = 5; ****P < 0.0001).

**Figure 5:**
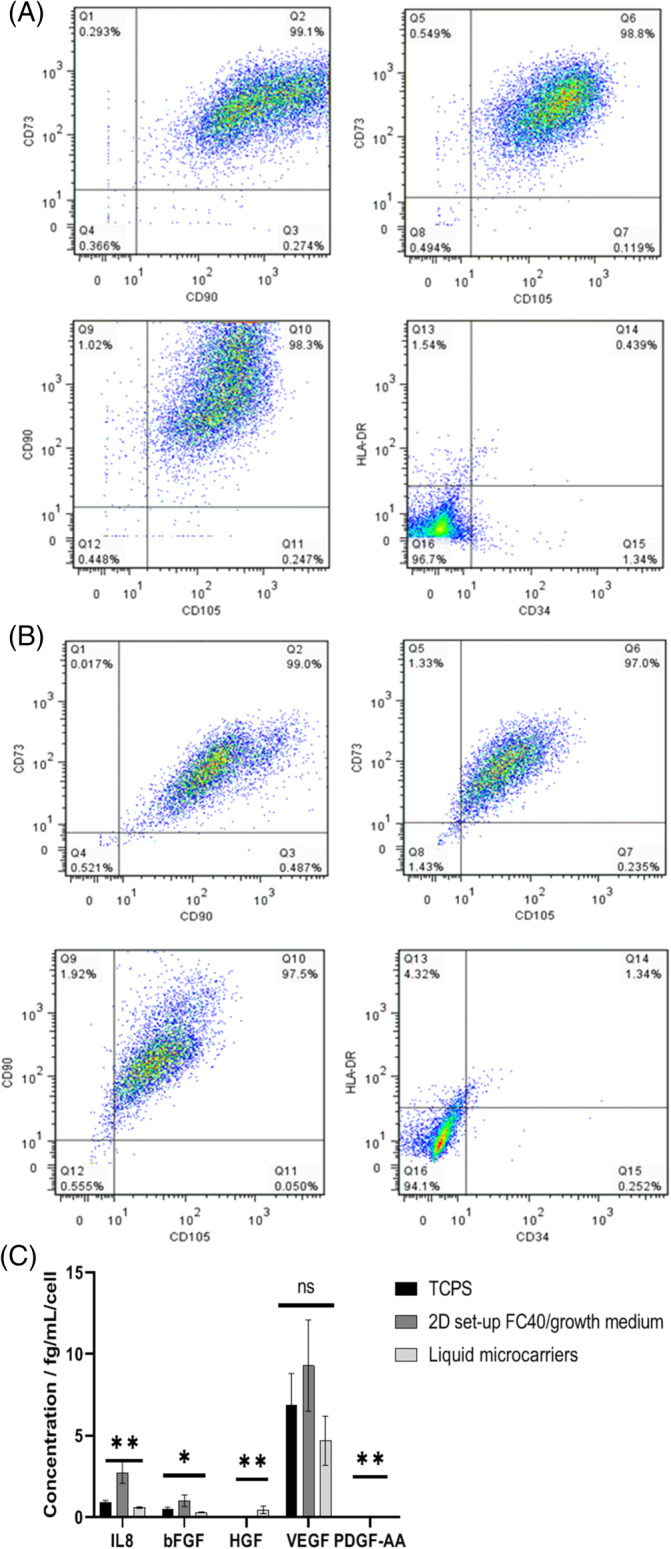
Multiparameter flow cytometry assessment of hMSC2 donor line expression of CD73, CD90, CD105, HLA‐DR and CD34 (A) pre‐expansion (TCPS) and (B) post‐expansion on the liquid microcarriers. (C) Pro‐angiogenic growth factor profile per cell at day 6 in culture for hMSC2 donor line cultured on TCPS and liquid microcarriers. Data are shown as mean ± SD (*n* = 3; **P < 0.001; ns, non‐significant.

Cell exposure to the culture environment in a stirred bioreactor may have a detrimental effect on cell quality. As such, cell characterisation needs to be performed before and after bioprocessing to ensure that none of the steps taken impacts cell quality negatively. Guidelines for hMSC characterisation were previously published by Dominici *et al*. and included replating on TCPS, differentiation towards three lineages (adipogenic, osteogenic and chondrogenic), clonogenicity assessment and cell surface marker expression.^30^ For assessing cell quality post expansion on the liquid microcarriers, the cells were harvested initially by enzymatic means. Figure 4(A) shows hMSC2 donor line morphology after replating on TCPS. Figure 4(B) shows the differentiation potential before and after culture on the liquid microcarriers. Differentiation to adipocytes was deemed successful both pre and post culture on the liquid microcarriers due to the presence of lipid vesicles stained in red, which are typical of adipocytes.^31^ Differentiation to osteogenic lineage was also deemed successful, as bone mineralisation was visibly stained in black, while alkaline phosphatase expression, which is specific to osteocytes,^32^ was evident through the red staining. Similarly, chondrogenic differentiation was successful as micromasses were formed and stained in blue, indicating strong formation of glycosaminoglycans representative of cartilage.^33^ Clonogenicity potential was also assessed before and after expansion on liquid microcarriers (Fig. 4C). CFU‐f efficiency was found to be significantly lower post expansion (****P < 0.0001). However that difference could be attributed to the increase in cell passage number. A panel of five cell surface markers was assessed by flow cytometry. Figure 5(A) shows the cell surface marker expression before expansion and Fig. 5(B) after expansion on the liquid microcarriers. In both scenarios, expression of CD73, CD90 and CD105 was above 97% positive, while expression of CD34 and HLA‐DR was below 2% positive. Post expansion on the liquid microcarriers, this expression profile was retained (Fig. 5B).

There are many physiological processes that hMSCs are believed to influence through paracrine effects.^34^, ^35^ Angiogenesis is one such process. Pro‐angiogenesis growth factors secretion was assessed before (when cultured on TCPS) and after culture of hMSC2 donor line on the liquid microcarriers (Fig. 5C). There was a significant difference (**P < 0.001) between the interleukin‐8 (IL‐8) levels per cell produced by the hMSC2 donor line at day 6 when cultured on TCPS (0.943 ± 0.09 fg mL^−1^ per cell) compared to the liquid microcarriers (0.602 ± 0.05 fg mL^−1^ per cell) (Fig. 5C). IL‐8 is a key factor in capillary tube organisation, while promoting higher proliferation rates of epithelial cells.^36^ The higher IL‐8 levels per cell produced when the cells were cultured on TCPS could indicate an increased capacity to form capillary tubes and an increased proliferation rate on that substrate. The former has not been verified here. However, the lower IL8 levels correlate with a lower proliferation rate when cultured on liquid microcarriers. Thus, it could be hypothesised that these lower levels are linked to the formation of aggregates towards the later stages of culture (Fig. 3).

VEGF is another factor that was measured here and it is a potent mediator of angiogenesis, known to induce growth of blood vessels and to stimulate migration of endothelial cells.^37^ VEGF was produced in similar levels regardless of the substrate used (*P* > 0.05) (Fig. 5C). HGF is a regulator of cell growth, motility and morphogenesis,^38^ and it has also been found to stimulate the expression of a number of other growth factors and receptors, including VEGF.^35^, ^39^ No levels of HGF (0 ± 0 fg mL^−1^ per cell) were measured on TCPS, but some HGF (0.463 ± 0.231 fg mL^−1^ per cell) was recorded when cultured on liquid microcarriers. However, the amount was not significant (*P* > 0.05) (Fig. 5C). Here, the VEGF level at day 6 was not significantly different (*P* > 0.05) on any of the tested substrates, despite the increased level of HGF on liquid microcarriers.

bFGF was also measured. It is known to act similarly to VEGF and to mediate proliferation, migration and differentiation of epithelial cells.^40^ There was no significant difference (*P* > 0.05) in the bFGF levels produced by the hMSC donor line 2 at day 6 when cultured on TCPS or the liquid microcarriers (Fig. 5C). However, the bFGF levels were slightly lower on liquid microcarriers (0.307 ± 0.019 fg mL^−1^ per cell) compared to TCPS (0.498 ± 0.12 fg mL^−1^ per cell). It is worth noting that this result was somewhat expected as, at the end of hMSC culture on the liquid microcarriers (Fig. 3A), there was a decrease in cell number caused by the formation of cell aggregates due to the contraction of the interface between the two phases, which produced a decreased cell proliferative rate.

### Non‐enzymatic cell harvest assessment

The dispersions produced with the FC40 perfluorocarbon and growth medium were found to be stable for at least 5 days when stored at 4 °C, which was unexpected given that surfactants were not used within the formulation. Only after 5 days of storage, some signs of droplet coalescence were observed. Surfactants are typically used to produce stabilised dispersions of very fine drops (emulsions). Here, the drops in these stabilised dispersions are rather large compared to those typically found in emulsions. However, it can be hypothesised that the increased stability of the perfluorocarbon droplets was determined by the steric stabilisation caused by the proteins found in serum. Proteins are amphiphilic molecules and have been used before for stabilising oil‐in‐water emulsions.^41^ The hypothesis is that, once absorbed on to the interface, protein unfolding takes place, thus leading to the hydrophilic groups protruding into the aqueous phase (e.g., growth medium), resulting in droplet stabilisation by steric interactions. This hypothesis was tested and confirmed in our previous study.^26^ In that study, although perfluorocarbon droplet coalescence could not be achieved under dynamic conditions, it was successful when static, combined with multiple D‐PBS washes. Here, static coalescence was exploited to establish non‐enzymatic cell harvest.

**Figure 6:**
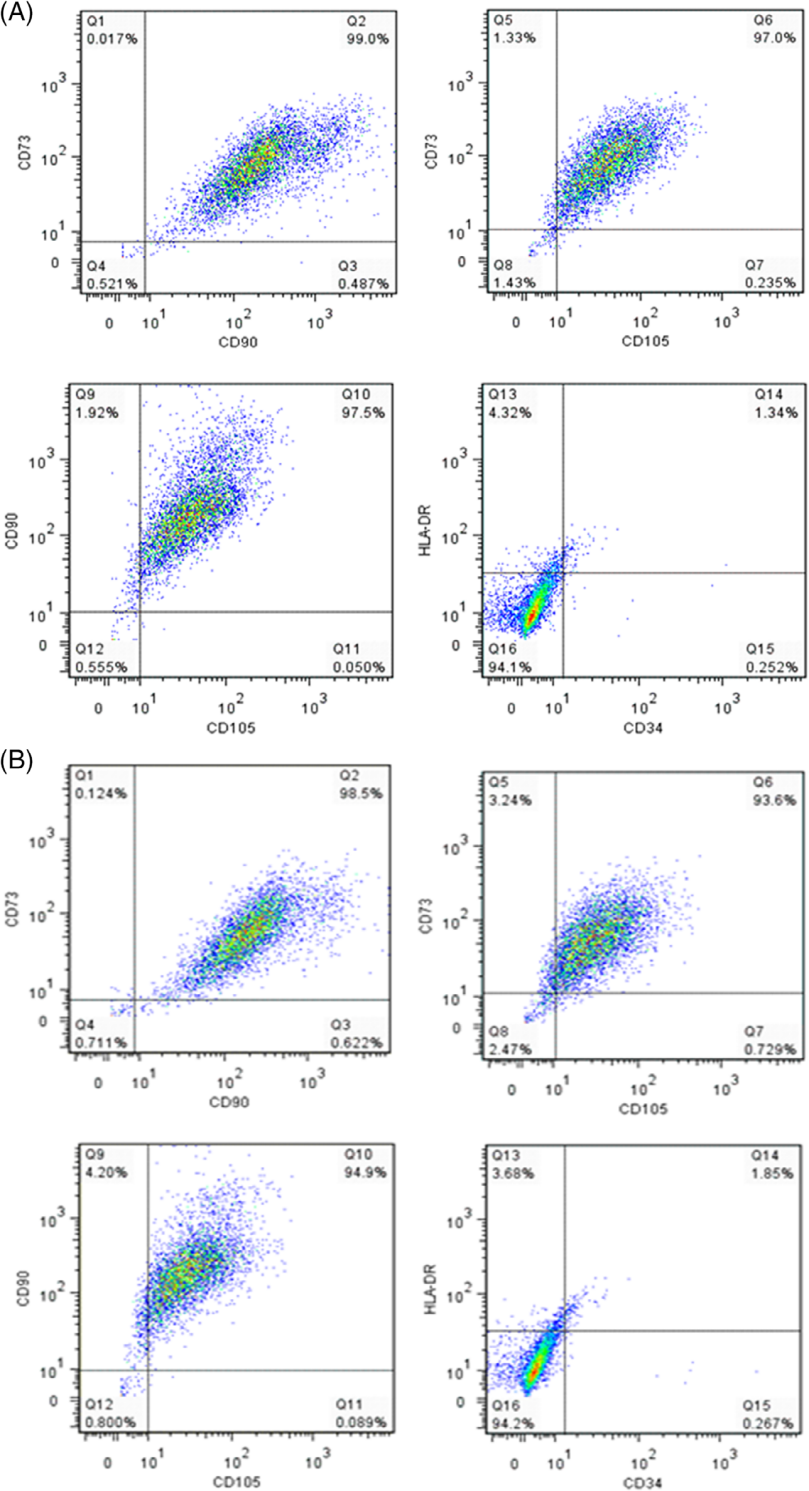
Multiparameter flow cytometry assessment of hMSC2 donor line expression of CD73, CD90, CD105, HLA‐DR and CD34: (A) before and (B) after inducing coalescence of liquid microcarriers.

**Figure 7:**
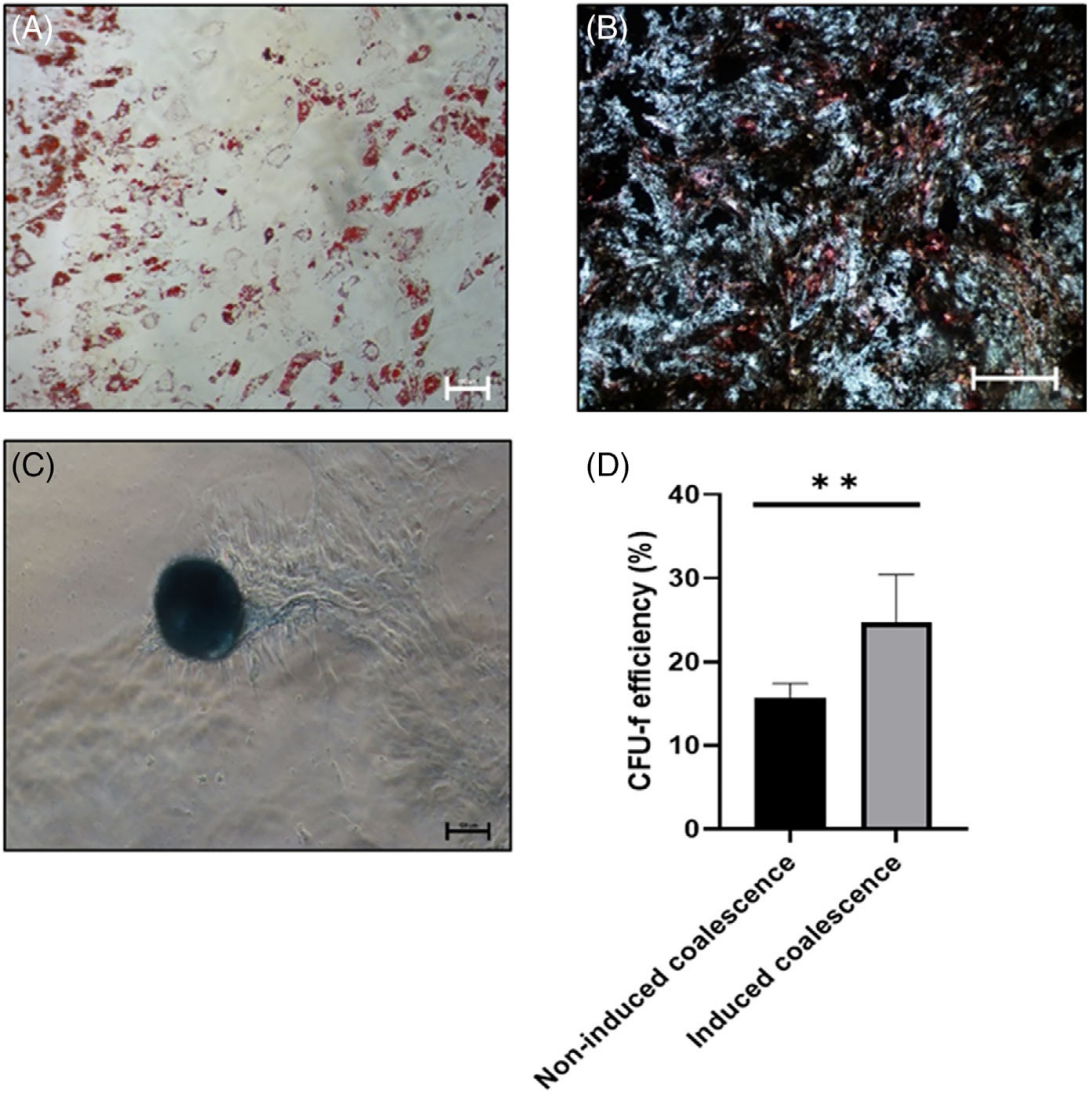
Differentiation post‐inducing coalescence of liquid microcarriers: (A) adipogenic; scale bar represents 100 μm; (B) osteogenic; scale bar represents 500 μm; (C) chondrogenic; scale bar represents 100 μm; (D) CFU‐f efficiency. Data are shown as mean ± SD (*n* = 5).

After four consecutive D‐PBS washes, complete droplet coalescence was achieved, which resulted in separation of the two phases and accumulation of the released cells to the interface, from where they were collected by aspiration with a sterile Pasteur pipette. The harvested cells were characterised and the results are shown in Figs 6 and 7. Post coalescence, the cells retained the high expression of CD73, CD90 and CD105 (>93%), while only a small proportion of cells (<3%) were HLA‐DR and CD34 positive (Fig. 6B). This expression was comparable to that seen pre coalescence (Fig. 6A). Differentiation potential towards the three lineages – adipogenic (Fig. 7A), osteogenic (Fig. 7B) and chondrogenic (Fig. 7C) – was retained post‐droplet coalescence. CFU‐f efficiency was found to be significantly higher (**P < 0.001) post‐droplet coalescence than by means of enzymatic harvesting from the liquid droplets (Fig. 7D). Non‐enzymatic means for harvesting cells from substrates such as temporary liquid microcarriers could be highly advantageous for the previously mentioned reasons and could lead to simplified manufacturing processes for cells for therapies.

## CONCLUSIONS

The prospect of a temporary microcarrier that can allow cell expansion and, when required at the end of culture, permit non‐enzymatic cell harvest, is an exciting one. This study describes the use of such temporary microcarriers represented by stable perfluorocarbon droplets dispersed in a serum‐based cell culture medium. These liquid microcarriers can be produced *in situ* in the bioreactor at any scale by applying mixing to break down the perfluorocarbon into droplets and disperse them in the medium. The proteins present in serum will then stabilise these droplets and, at the same time, condition them for cell culture. This is a simple and inexpensive system that can be tailored to the needs of culture. The mixing applied and ratio of perfluorocarbon‐to‐medium will dictate the size and size distribution of the liquid microcarriers.

In this study, hMSCs were used to test the suitability of the liquid microcarriers for culture. However, this approach could potentially be suitable for the culture of any adherent cell type. When tested with hMSCs, the cells successfully attached and grew on the liquid microcarriers, while, very importantly, retaining their quality post expansion and post non‐enzymatic harvest, achieved by inducing droplet coalescence through simple steps such as washes.

This study was carried out in a serum‐based medium. However, the manufacture of clinically relevant hMSCs for therapy will require the use of serum‐free and xeno‐free media. To overcome this challenge, our proposed liquid microcarriers could still be prepared in alternatives to serum such as human platelet lysate, which is still protein rich and could be used in the formulation of the two‐phase system without affecting the stability of the liquid microcarriers.

## CONFLICT OF INTEREST

The authors declare that there is no financial or commercial conflict of interest.
